# A Qualitative Study of Women’s Lived Experiences of Conflict and Domestic Violence in Afghanistan

**DOI:** 10.1177/1077801220935191

**Published:** 2020-07-06

**Authors:** Jenevieve Mannell, Gulraj Grewal, Lida Ahmad, Ayesha Ahmad

**Affiliations:** 1 UCL Institute for Global Health, London, UK; 2 Independent Consultant, Kabul, Afghanistan; 3 St George’s, University of London, UK

**Keywords:** domestic violence, Afghanistan, lived experience, patriarchy, armed conflict

## Abstract

This article empirically explores women’s lived experiences of domestic violence and conflict in Afghanistan. A thematic analysis of 20 semistructured interviews with women living in safe houses produced three main themes about the relationship between conflict and domestic violence: (a) violence from loss of patriarchal support, (b) violence from the drug trade as an economic driver, and (c) violence from conflict-related poverty. We discuss the bidirectional nature of this relationship: Not only does conflict contribute to domestic violence, but domestic violence contributes to conflict through justifying armed intervention, separating women from economic and public life, and perpetuating patriarchy.

## Introduction

Violence against women is a global problem that has severe implications for both physical and mental health (García-Moreno et al., 2005). The United Nations has defined violence against women as

any act of gender-based violence that results in, or is likely to result in, physical, sexual, or mental harm or suffering to women, including threats of such acts, coercion or arbitrary deprivation of liberty, whether occurring in public or in private life. (A/RES/48/104. Declaration on the Elimination of Violence Against Women [EVAW], 1993)

In Afghanistan, such gendered forms of violence against women may include domestic violence perpetrated by both husbands and mothers-in-law (Jewkes et al., 2019), forced marriage, honor killings, rape by strangers, body mutilation, and forced prostitution (Ahmad & Avoine, 2016; Gibbs et al., 2019; Mannell et al., 2018). Studies from other settings have shown that living in conflict settings significantly increases the likelihood that women will experience gendered forms of violence (Kelly et al., 2018). This article investigates women’s lived experience of this association between gendered forms of violence and conflict in Afghanistan to draw conclusions for addressing violence against women in conflict settings more broadly.

In this article, we use the term domestic violence to encompass gender-based violence perpetrated against women by an intimate partner (e.g., a husband) and perpetrated by other members of a woman’s household, including mothers-in-law (who often live with women in extended family households in Afghanistan). The research question framing our study is as follows:

**Research Question 1:** In what ways do the lived experiences of Afghan women contribute to our understanding of violence against women during conflict?

In asking this question, we are drawing on the phenomenological study of lived experience, which takes account of the meaning individuals attribute to particular experiences at particular moments in time (Manen, 2016). This provides the opportunity for a rich account of women’s experiences of domestic violence during a time of prolonged conflict in Afghanistan. The article begins with a summary of the current state of the literature on domestic violence and conflict followed by an overview of the current conflict and women’s lives in Afghanistan. We then outline the methods used for our empirical study and our findings.

### Current Literature on Domestic Violence During Armed Conflict

There is a growing body of literature on how armed conflict perpetuates gender-based violence and creates an environment of increased hostility for women. Armed conflict is defined in this literature as violence between state and/or nonstate actors that leads to deaths in a population (Allansson et al., 2017). The association between increased domestic violence and armed conflict has been well established, as have the serious consequences this has for women’s mental and physical health (Gupta et al., 2014; Rubenstein et al., 2020; Stark & Ager, 2011). Relatively higher rates of domestic violence also appear postconflict (Horn et al., 2014; Saile et al., 2013), with women living in conflict-affected areas with high fatalities being more likely to experience intimate partner violence postconflict (Kelly et al., 2018), and men who either witness or partake in violence during conflict more likely to be violent to their intimate partners (Clark et al., 2010). Men who experience political violence by the state are also more likely to perpetrate violence against both family members and intimate partners (Clark et al., 2010; Gupta et al., 2009, 2012; Sousa et al., 2018).

A related body of literature has focused on and contributed to an international recognition of rape as a “weapon of war” and new awareness of sexual violence during conflict perpetrated by the army, paramilitaries, and peacekeepers (The PLoS Medicine Editors, 2009; Kivlahan & Ewigman, 2010; Koss et al., 1994; Lancet, 2004; Mer & Flicourt, 2015; Nowrojee, 2005; Sharlach, 2000). However, evidence suggests that sexual violence perpetrated by strangers during armed conflict is not as prevalent as intimate partner violence in many conflict-affected settings (Stark & Ager, 2011). Research from Côte d’Ivoire similarly indicates that domestic sexual violence may have been more prevalent than nonpartner rape during the conflict (Hossain et al., 2014).

The increased risk of domestic violence in conflict and postconflict settings has individual and structural explanations, which are interrelated. At an individual level, conflict is seen as having enduring impacts on the mental health of both men and women, with perceptions of these impacts being associated with both rape perpetration and partner violence (Jewkes et al., 2017). Evidence from refugee camps in Afghanistan and Pakistan highlights how stress due to conflict and poverty can lead to violence by men in family settings as a means of regaining a sense of power and control (Hyder et al., 2007). Similarly, the inability of men to fulfill the culturally defined role of “provider” in unstable or impoverished settings can contribute to domestic violence when wives ask for something that men feel unable to provide (Horn et al., 2015). Structurally, conflict at a regional or national level can be understood as a struggle between individuals or groups over status, power, ideology, and resources, which manifests at the individual level in men trying to achieve idealized and impossible forms of masculinity through the use of violence (Baaz & Stern, 2009). Gender norms that condone acts of violence against women are often intertwined with gender norms that help individuals establish psychological security and stability during, and soon after, times of crisis (Guruge et al., 2017; Horn et al., 2015).

Recent literature on domestic violence in Afghanistan has highlighted the clustering of violence that takes place within families, and the ways in which extreme forms of violence, such as honor killings, are often an extension of the use of violence against women more broadly (Gibbs et al., 2019). Women in Afghanistan are also more likely to experience physical violence by their mothers-in-law if they are also being abused by their husbands, providing further evidence of the clustering of violent acts within households (Jewkes et al., 2019). While critically important in developing understandings of violence against women at the household level, there is little evidence of how the conflict itself may be acting as a driver of violence against women in Afghanistan or the ways in which this may be fuelling household violence in this context. This has been explored in other conflict settings and in Afghan refugee populations as summarized above, but not in Afghanistan, which has seen one of the longest armed conflicts in recent times.

To help fill this gap, we examine the intersection between women’s lived experiences of the armed conflict and domestic violence evident in the results of an empirical qualitative study conducted with women living in safe houses—shelters for survivors of gender-based violence—in Kabul. This contributes to the current literature by interrogating the relationship between conflict and domestic violence in Afghanistan from the perspective of women living at the crossroads of these two types of violence. This has broader implications for understandings of the relationship between armed conflict and violence against women, which are discussed in light of our findings.

### Case Study: Women and the War in Afghanistan

Following the targeted attacks of 9/11, the United States invaded Afghanistan in an effort to destroy the Taliban movement as a “protectorate of Islamic extremism,” leaving most Afghans without food or proper housing (Cooley, 2002). A strong emphasis placed on the liberation of women from the oppressive regime was used as a justification for the initial intervention (Dossa, 2013). However, this justification for intervention was entirely undermined by support by the United States and other international actors for fundamentalist warlords, the Mujahidin, who came to power in the months following the Taliban’s defeat. The fact that the Taliban government was made up of war criminals and perpetrators of sexual violence was highlighted at the time in reports by Amnesty International (1995) and Human Rights Watch (2005), but widely ignored by international governments. A similar situation persists today, significantly undermining efforts to establish democracy and a women’s rights movement, due to the presence of Mujahidin leaders and their commanders in positions of power including as Ministers, governors, and members of parliament. Recent reports continue to highlight the perpetuation of acts of violence against women in Afghanistan by these warlords (Human Rights Watch, 2015).

After over a decade of U.S. military involvement, the United States announced it would withdraw combat forces from Afghanistan following peace discussions between the Afghan government and the Taliban in 2014, with several reversals on this commitment, a continued U.S. presence, and persistent bomb attacks on civilians to date (Rosenberg & Shear, 2017). Afghanistan’s opium trade has further exacerbated the conflict, and has grown in recent years with eradication efforts decreasing by 91% in 2016 due to security concerns (UN Office on Drugs and Crime, 2016). Heroin is believed to fund the majority of the Taliban’s activities, as well as causing addiction among local men (Cottler et al., 2014). Poverty, food insecurity, and unemployment all push men toward the opium trade. The ongoing conflict and patriarchal norms exacerbate the situation as men are expected to be the ultimate breadwinners, or *nan avar*, and provide for their family by any means necessary, especially in conservative rural settings (Echavez et al., 2016; Nawa, 2011).

The situation for women in Afghanistan severely undermines protections from violence. According to the 2009 law on EVAW, women cannot be forced into marriage, undergo underage marriage, have the right to education and work, and can report abuse to the police resulting in the punishment of the offender. Moreover, women are rarely passive victims, and have played an active role in resisting the restrictions they experienced during the time of the Taliban, developing secret networks of solidarity and sometimes turning their homes into schools for girls’ education (Povey, 2003). In practice, however, women face numerous obstacles such as police corruption, medical practitioners who are fearful of getting involved in family disputes, and a lack of economic independence that severely limits opportunities for women to leave violent situations. Women’s lives outside of the home are controlled by a *mahram*, a male relative, as part of a system of patriarchal guardianship put into place by the Taliban, whereby women are often required to have a male relative accompany them when leaving the home in order to preserve their honor. Visiting a male doctor is outside of current cultural norms, a remaining product of the enforced Taliban regime, making health care difficult to access as there is a severe lack of female doctors (Stokes et al., 2016). Imprisonment or honor killings are integral to circumventing expected social norms around marriage or household duties for a daughter such as when women refuse to marry the man their father or guardian has chosen, and elopement, sex before marriage, or suspected adultery are also often punished. According to Human Rights Watch (2017), the Supreme Court ruling in December 2015 banning the imprisonment of women for running away from their families has not had much effect, as women still need to visit a medical provider, the police, or the house of a close male relative. As such, women face numerous difficulties when leaving forced or abusive marriages.

## Method

Qualitative data were collected from March to May 2017 from safe houses for women who had experienced domestic violence in Afghanistan. Twenty semistructured interviews were conducted by the third author with a convenience sample of women living in two safe houses operated by a local nongovernmental organization (NGO) at the time of data collection.

Conducting research in safe houses for women experiencing domestic violence in Afghanistan understandably raises numerous ethical challenges. These were carefully considered by the project team, and measures put in place to mitigate potential risks to the participants and project team members at all stages of the project. The most significant concern was ensuring the psychological safety of the women we interviewed. We mitigated this risk by not directly asking women any questions about the violence they had experienced, although these experiences did emerge in the majority of the interviews. We also ensured that the NGO operating the safe houses had psychological support staff on hand who could talk to the women following their interview in case of any psychological distress arising from the interview process itself. The psychological safety of the project team was protected through regular open discussions about the potential for vicarious trauma (Dickson-Swift et al., 2007; Ellsberg et al., 2001), awareness of the symptoms, and knowledge about the possibilities for referrals to psychological support services by any of the team members. Additional ethical concerns included the protection of the women’s identity and anonymity of the safe houses themselves. For these and other ethical concerns, we referred to the World Health Organisation’s (WHO’s) guidelines for conducting research on violence against women (WHO, 2016). We obtained ethical approval from both UCL’s Research Ethics Committee (reference number 6228-002) and the NGO operating the safe houses in Afghanistan prior to the start of data collection.

### Recruitment and Sample

Initial agreement for the data collection was obtained by the third author (LA) through discussions with the NGO, with whom she had previously done research. LA visited the safe houses on several occasions to carry out the interviews. The population of the safe houses changes as women arrive and leave with different personal circumstances, and on each visit, LA asked the women whether they would like to participate in an interview about storytelling. Those who agreed were given an information sheet, were explained the purpose of the study and that they would be recorded, and asked to give their verbal consent once the tape recorder was switched on. A total of 20 women agreed to be interviewed. The age range of these women was between 18 and 41 years. They had been living in the safe house for between 1 month and 4 years, and had come to the safe house from different regions of Afghanistan.

### Data Collection

The interviews were conducted in either Pashto or Dari. Women were asked a series of semistructured questions starting with “Can you tell me about a story that is meaningful to you?” They were then asked broad open-ended questions about violence against women in Afghanistan and about how easy it was for women to tell their stories in Afghan society. The open-ended nature of our question guide meant that many of the women talked about their experiences of violence in the context of their broader life narrative, their experiences of growing up, and of the war and its impacts on their lives. Although we did not specifically ask about the conflict, this was a dominant theme in the women’s stories and a defining feature of their experiences. Forty years of continuous conflict in Afghanistan makes this a defining feature of many women’s lived experiences. The interviews were transcribed and translated into English by LA. The complete transcripts were then encrypted and transferred to the U.K.-based project team for analysis through an in-person visit to Afghanistan.

### Data Analysis

The interview transcripts were analyzed both inductively and deductively using thematic analysis procedures outlined by Attride-Stirling (2001). After initial review of the interviews, a preliminary coding structure was discussed and formed by the first and second authors. Interview transcripts were carefully examined and coded using NVivo, with additional codes being added to the coding structure as they emerged from the analysis. Upon reaching consensus on the coding structure, related codes were grouped into organizing themes, which were then examined contextually using previously published literature on domestic violence. Through this process, a thematic network was formed with conflict at the center of the thematic map and overarching subthemes of patriarchal support, the drug trade, and conflict-related poverty flowing from this central concept.

### Limitations of This Study

Our study was carried out with women living in safe houses in Kabul, which offers a particular perspective on the conflict and experiences of violence. Although Kabul is an urban environment, women and the stories they told came from different regions of Afghanistan due to a lack of available resources outside of the capital. However, women who live in safe houses have successfully navigated a limited system of support for women experiencing violence and have chosen to leave their families, stepping outside of socially accepted norms. Our data, therefore, do not capture the perspective of the majority of Afghan women who remain in violent relationships.

Our study was also limited by a convenience sample of participants who happened to be living in the safe houses at the time of data collection. This sampling method has a number of limitations in terms of the possibilities that exist for ensuring a diversity of perspectives among the participants. The sample size was also fairly small (*n* = 20) for these same reasons, which again limits the variety of perspectives that may have been captured. Additional themes about the relationship between conflict and domestic violence may have arisen in a larger sample. We tried to ensure the comprehensiveness of our data through careful analysis, and searching for evidence of data saturation (e.g., themes were shared across multiple interviews and no new themes emerged in the final stages of analysis). Although we do feel that saturation was achieved in our study, a wider study across different provinces of Afghanistan would be valuable for the future.

## Findings

In the interviews, women described their experiences of violence in relation to their broader life story, the history of their family, and the consequences of the conflict for their lives. Three main themes emerged from the analysis of women’s stories, which illustrate the relationship between conflict and domestic violence in Afghanistan: (a) violence resulting from the loss of patriarchal support, (b) violence resulting from the drug trade as an economic driver of conflict, and (c) vulnerability to violence resulting from poverty brought on by conflict.

### Violence Resulting From the Loss of Patriarchal Support During Conflict

The conflict in Afghanistan has led to a significant loss of life. As a patriarchal society, the loss of a husband, father, or brother because of the ongoing conflict can have severe implications for women’s lives. Under the social system of guardianship, women are rarely able to make their own decisions, including about marriage. The loss of a male guardian whom they trust, therefore, can leave them exposed to decisions being made on their behalf with little consideration of their own hopes or desires, as in the case described by one of our participants:

I accepted to get marry with the person my father engaged me. He was nice man. He was nice, but my brothers started to interfere in my life. . . . Then I lost my fiancé in a bomb blast . . . they [her brothers] engaged me again with another man. I didn’t know him. It was against my will. (B.B., age 27, 4 years in safe house)

Another participant described how her mother was forced to marry her husband’s brother after the death of her husband as a result of the conflict:

My mother suffered so much. She lost my father. My father was a teacher. The Taliban threatened him several time and asked him to stop teaching. However, my father was a nice and educated man, and he continued to teach children of my village. Twelve years ago, he was shot dead. After that my uncle (my father’s brother) forced my mother to marry him. All of this was pain for her. She didn’t want to get married again. She wanted to be with us. (H, age 18, 10 months in safe house)

The ways in which the death of men during the conflict in Afghanistan has led to violence against women in the form of forced marriage is evident in both of these examples from the interviews, as is the traumatic impact this loss and its violent consequences can have on both women and children’s lives.

Women also discussed how interpersonal conflicts in their families and communities led to the loss of a patriarchal guardian, which in turn contributed to their own individual experiences of violence; for example,

My family suffered so much. They [her uncle and his friends] killed my father, then my older brother and then the younger one. . . . My uncle forced me and my sister to marry his sons. It happened against our will. My uncle did all these things to grab our father’s fields. I didn’t want to marry his son, but they beat me. Now I am pregnant. They beat me and broke my leg while I was pregnant. (K.M., age 19, 4 months in safe house)

This example demonstrates how it is not only national conflict that contributes to violence against women by killing male guardians during acts of war, but also conflict at community and family levels that becomes possible when systems of governance and legal protection are absent or lacking.

### Violence Resulting From the Drug Trade as an Economic Driver of Conflict

The drug trade that has fuelled the conflict in Afghanistan was frequently discussed by the women we interviewed as having serious consequences for their lives. Many of the women’s stories had to do with violence perpetrated by male guardians (husbands or fathers) who were addicted to drugs such as heroin or hashish. Some women were forced into marriages with known drug addicts; for example,

My sister and I suffered a lot. We lost our mother, and my father couldn’t take care of us so he engaged me to our neighbor. He was working in a bakery. He was a drug addicted. My father got money from him without thinking about my life. My father forced me into this marriage. He didn’t work, and the small salary he had, he used to buy drugs. I didn’t have money. Now I have two children, both girls. When I was pregnant I couldn’t eat good food [because of lack of money]. (M, age 19, 7 months in safe house)

Two forms of violence are evident in this example: first, the forced marriage, and second, the economic violence of not being given money or food by her husband who was addicted to drugs. The role of drug addiction in contributing to dire economic situations for women was a prevalent theme in the interviews. Several women described how drug addiction led to significant psychological and physical abuse:

My mother is tormented like me. My father is an addicted man. He was always angry and shouted at my mom. My mother was crying, my father took money from her to buy drugs. If my mother didn’t give him, he would beat her. (N.J., age 20, 1 year in safe house)

For other women, the root cause of the violence was not only about the addiction but also about the role the drug trade has played in creating powerful and abusive men:

My sister suffered in her life. She married when she was only 15. Her husband is a warlord. He is addicted and beats her. Sometimes offering her to his friends. My sister doesn’t want it. She always cries. (S., age 19, 6 years in safe house)

The consequences of drug trade for different forms of violence including sexual, psychological, and physical were evident across the interviews. Women’s stories also highlight how the relationship between drugs and violence, similar to forced marriage, is deeply embedded within the patriarchal system of Afghan society. For instance, one of our participants had been exchanged in marriage to pay a drug-related debt for her brother:

My brother hurt me and my sister and my mother. He is working with a drug smuggler. Many times he was jobless. He just got money from certain people to survive. All the people who have given him money, asked for their money back, but my brother didn’t have the money. The smuggler, who is 50 years old, suggested that he would take care of all his debts, if he gave his sister (who is me) to him. (H, age 18, 10 months in safe house)

It is the limited value of women’s lives within a patriarchal society that creates the possibility of trading a woman’s life to pay a drug debt. Funding conflict through the sale of drugs has clear implications for violence as it increases the number of individuals dependent on drugs, and gives power to individuals who exist outside of legal frameworks. However, in a patriarchal society where women have little power, they become increasingly vulnerable to experiencing the negative impacts of this relationship between conflict and drugs.

### Vulnerability to Violence Resulting From Poverty Brought on by Conflict

The ongoing conflict has significantly reduced the economic stability of the majority of households in Afghanistan, plunging many families into severe poverty. This had consequences for the lives of the women we interviewed, who spoke about how the poverty of their families as they were growing up had directly increased their risk of experiencing violence later in life. This is particularly evident in the story of K who lost her father at a young age and was subsequently forced into beggary and an abusive marriage with one of her mother’s relatives. In the interview with K, she discusses poverty as the reason why her father joined the Taliban:

I remember my mother telling me the story of war during the time of Taliban. It was a bad time for all people who were living in Kandahar. At that time my mother got married, my father lost his job. Then he started working with the Taliban as a cook. They didn’t give him salary so he used to bring some leftover food for us. My mother said at that time life was difficult but it was fine, at least my father was alive to work and providing us with food. During the collapse of Taliban, Kandahar was bombed by an American airplane, and my father and some other men were killed. (K, 18, 2 months in safe house)

In K’s story, poverty and instability contributed to her father’s engagement in the conflict as a source of support, putting his life and the future stability of his family at risk. In a similar way, in another interview, QG tells a story of how poverty limited her family’s ability to make choices that could protect her from violence:

Since I can remember, my father had been sick. He couldn’t walk or move his hands. I was 12 years old when he died. The story of my father is that when I was 9 months old, he went to do his military duty for two years. During those two years, he got rheumatism. He was sick but we couldn’t treat him properly, because we were poor and lived in a remote village in the north of Afghanistan. I was 15 years old when my uncle engaged me to his son. His son belonged to the Jihadi party (Jamit-e-Islam). He is very violent and addicted to hashish. He used to beat me from the very first days of our marriage. He accused me of going with other men. He threatened me with a gun and I was afraid. Some years back, he had killed a man. (QG, 41, year and a half in safe house)

In this case, poverty reduced the ability of QG’s family to protect her from a violent marriage. By perpetrating instability and the formation of new political groups, the conflict offers new options for employment (as in both QG’s and K’s stories), but these options are tainted by conflict-related violence. Although those with power and economic resources may be able to navigate the instability of conflict, the poor are left even more vulnerable to the deficient set of options it offers them.

## Discussion and Conclusion

These interviews with women provide an account of the lived experience of violence among women during the conflict in Afghanistan. Our findings are similar to findings from other conflict settings about the ways in which poverty and economic stress translate into a need by men to exert control over women (Hyder et al., 2007), and the role of drugs and alcohol as risk factors for domestic violence (Brisibe et al., 2012; Cunradi et al., 2012; Field et al., 2004). In focusing on women’s lived experiences of conflict and domestic violence, we have also been able to trace the interconnections between the “public” spaces of the conflict and the “private” spaces of the home, and highlight the nuanced interconnections within and between these spaces. The conflict contributes to the death of family members, the proliferation of the drug trade, and poverty and instability in ways that lead to acts of domestic violence including forced marriage, neglect, and severe physical and psychological abuse. The conflict, therefore, is not something that happens exclusively in the outside environment with women living protected lives inside the home, but an experience that permeates every aspect of women’s lives and their experiences of violence.

This resonates with long-standing feminist discussions about the false separation of public and private spheres, with the public sphere often depicted as the rightful place of men and the private household as the rightful place of women (MacKinnon, 1989). Feminists argue that most social practices do not belong to either the private or public sphere but rather economic exchanges, the role of emotions in human relationships, power hierarchies, and legal changes; in other words, political acts not only are similar across public and private spaces but also interact in mutually reinforcing ways (Gal, 2002). Our findings support these arguments and highlight how the distinction between public and private spheres is an ideology that reaffirms patriarchal social relationships and limits the role of the state in protecting the rights of women in the home (Ackelsberg & Shanley, 2018; Rose, 2017). The patriarchal system that confines women to the home in Afghanistan relies on clear divisions between public and private spheres, and gendered notions of who rightfully belongs in these spaces. The conflict is often used as a means of justifying the need for women’s safety and home as a “safe” space in ways that obscure how these patriarchal ideologies create new vulnerabilities for women during conflict.

Moreover, the Afghanistan government’s consideration of domestic violence as a public matter of concern has been haphazard. Government actors, NGOs, and activists continue to dispute whether domestic violence is a public issue requiring state involvement and legislation, or whether it should be protected as a private concern with no space for state intrusion (Ahmad & Avoine, 2016). The historically embedded perception of domestic violence as a private issue within Afghan society has meant that domestic violence receives very little attention in public spaces. In this way, the division between public and private spaces is used to justify nonaction or involvement of the government in what are perceived as “household” affairs, effectively meaning anything that affects women’s lives.

This ideological separation of private and public sphere as a means of justifying domestic violence and noninterference by the state is certainly not unique to Afghanistan. As in all societies, violence against women is the result of socially embedded gender norms and inequities (Anderson, 2005). Although Afghanistan has its own unique gender norms and structures, including male guardianship and *purdah* (the practice of secluding women from the gaze of men or strangers), these are only the mechanisms through which gender norms and structures manifest as violence against women in this context. Although the mechanisms may be different in other contexts, ideological divisions between private and public spheres often operate in a similar way by maintaining perceptions of domestic violence as a private act and, therefore, outside the reach of political interference or public intervention.

Although conflict increases the prevalence of domestic violence in most settings, the more novel finding arising from our study is the ways in which domestic violence can also perpetuate conflict. We argue that this appears to be happening in Afghanistan at three levels. First, domestic violence effectively separates women from both economic and public life in ways that contribute to widespread poverty and further economic instability. In many settings, including Sierra Leone, Liberia, and Rwanda, war itself can become an entry point for women into public spheres as they are required to take up work to support their families in the absence of male providers (Carlson, 2012; Horn et al., 2014). However, our findings from Afghanistan point instead to the increasing vulnerability of women who have lost their closest male relative in the conflict. Rather than becoming independent financial providers for themselves and their children, women are often transferred to other male guardians, either husbands or uncles, who may not have the same concern for their personal safety and well-being. This has an economic impact by virtually removing half of the population from paid work, as has long been argued by feminist economists (Kabeer & Natali, 2013). It can also have the effect of further impoverishing families who are already struggling as a result of the conflict by requiring them to support the female dependents of relatives killed during the conflict, often with dire consequences for these dependents. This contributes to a cycle whereby gender inequalities contribute to economic instability, which in turn drives conflict (Goodhand, 2003).

Second, domestic violence and its acceptance further perpetuate patriarchal social structures in ways that concentrate the power of warlords and criminality in Afghanistan. Patriarchal social structures that position women as commodities allow individuals to derive value from women’s bodies, including—as mentioned in our findings—through forced prostitution or dowry. In this way, the violence inflicted on women through exploitation and abuse becomes a commodity that others, primarily men, are able to use in struggles over power and resources as part of the conflict.

Third, the state of women’s health and rights in Afghanistan has been used as a means of justifying the need for international intervention. Although this was not the focus of our findings, it is important to acknowledge this component of the relationship between violence and conflict for the insights it provides to women’s lived experiences. Women’s lives in Afghanistan have been deeply affected by the proxy and antiterrorism wars carried out by foreign powers on Afghanistan soil for the past 40 years in ways that have magnified the violence they experience from men in their daily lives. Violence against women in this context is not only a manifestation of deeply embedded patriarchal systems such as guardianship and *purdah*, but the result of ongoing poverty and hardship, a lack of governance, and exposure to persistent conflict that has affected the region for decades. In this way, any claim by foreign governments or organizations that Afghan women need to be saved from Afghan men should be countered first by evidence that foreign actors have played a significant role in creating the problem in the first place.

In the Afghanistan context, listening to women’s lived experiences contributes to much-needed discussions about the complexities of conflict. The story of a woman’s life at home during war receives very little focus in the existing literature on Afghanistan, which instead has been dominated by narratives that link the liberation of an Afghan woman to the core of the conflict and efforts to denounce the Taliban. The veiled woman, the visible image of burka-clad women walking the streets of Kabul, have become worldwide symbols of oppression and victimhood, undermining women’s perspectives and deeper theoretical discussions of how conflict drives structural inequalities in ways that both perpetuate and feed from violence against Afghan women. In this article, we have provided an alternative narrative of how conflict and violence against women are deeply interrelated in Afghanistan, and the bidirectional nature of this relationship.
